# Identifying the needs of older people living with HIV (≥ 50 years old) from multiple centres over the world: a descriptive analysis

**DOI:** 10.1186/s12981-022-00488-7

**Published:** 2023-02-12

**Authors:** Tomás Martín Grosso, Diana Hernández-Sánchez, Gordana Dragovic, Marta Vasylyev, María Saumoy, José Ramón Blanco, Diego García, Tetiana Koval, Cora Loste, Tendayi Westerhof, Bonaventura Clotet, Omar Sued, Pedro Cahn, Eugènia Negredo

**Affiliations:** 1 Unidad de Ensayos Clínicos, Fundación Huésped, Buenos Aires, Argentina; 2 Laboratorio de Inmunología, Universidad Nacional de Luján, Buenos Aires, Argentina; 3 Lluita contra les Infeccions, Hospital Universitari Germans Trias i Pujol, Badalona, Spain; 4 Universitat Autònoma de Barcelona, Barcelona, Spain; 5 Department of Pharmacology, Clinical Pharmacology and Toxicology, Faculty of Medicine, University of Belgrade, Belgrade, Serbia; 6 Lviv Regional Public Health Center, Lviv, Ukraine; 7 HIV and STD Unit, Hospital de Bellvitge, Barcelona, Spain; 8 Infectious Disease Department, Hospital Universitario San Pedro - CIBIR, Logroño, Spain; 9 Adhara HIV/AIDS Association, Sevilla Checkpoint, Seville, Spain; 10 Department of Infectious Diseases, Poltava State Medical University, Poltava, Ukraine; 11 AIDS Research Institute-IRSICAIXA, Hospital Universitari Germans Trias i Pujol, Badalona, Spain; 12 AIDS Research Institute-IRSICAIXA, Hospital Universitari Germans Trias i Pujol, Universitat Autònoma de Barcelona, Badalona, Spain; 13 Universitat de Vic - Universidad Central de Catalunya (UVIC-UCC), Vic, Spain

**Keywords:** Older People Living with HIV, Aging, Health-Related Quality of Life, Patient satisfaction, Multicentre study

## Abstract

**Background:**

Older People Living with HIV (OPWH) combine both aging and HIV-infection features, resulting in ageism, stigma, social isolation, and low quality of life. This context brings up new challenges for healthcare professionals, who now must aid patients with a significant comorbidity burden and polypharmacy treatments. OPWH opinion on their health management is hardly ever considered as a variable to study, though it would help to understand their needs on dissimilar settings.

**Methods:**

We performed a cross-sectional, comparative study including patients living with HIV aged ≥50 years old from multiple centers worldwide and gave them a survey addressing their perception on overall health issues, psychological problems, social activities, geriatric conditions, and opinions on healthcare. Data was analyzed through Chisquared tests sorting by geographical regions, age groups, or both.

**Results:**

We organized 680 participants data by location (Center and South America [CSA], Western Europe [WE], Africa, Eastern Europe and Israel [EEI]) and by age groups (50- 55, 56-65, 66-75, >75). In EEI, HIV serostatus socializing and reaching undetectable viral load were the main problems. CSA participants are the least satisfied regarding their healthcare, and a great part of them are not retired. Africans show the best health perception, have financial problems, and fancy their HIV doctors. WE is the most developed region studied and their participants report the best scores. Moreover, older age groups tend to live alone, have a lower perception of psychological problems, and reduced social life.

**Conclusions:**

Patients’ opinions outline region- and age-specific unmet needs. In EEI, socializing HIV and reaching undetectable viral load were the main concerns. CSA low satisfaction outcomes might reflect high expectations or profound inequities in the region. African participants results mirror a system where general health is hard to achieve, but HIV clinics are much more appealing to them. WE is the most satisfied region about their healthcare. In this context, age-specific information, education and counseling programs (i.e. Patient Reported Outcomes, Patient Centered Care, multidisciplinary teams) are needed to promote physical and mental health among older adults living with HIV/AIDS. This is crucial for improving health-related quality of life and patient’s satisfaction.

## Background

During the last decades, remarkable improvements have been seen on the treatment landscape of HIV infection. Combined Antiretroviral Therapy (cART) has allowed controlling HIV infection with high efficacy and reasonably simple treatment schedules, in countries where treatment is widely available [1–4]. Consequently, the profile of persons living with HIV (PLWH) has been shifted towards a tendency of increasing age [5, 6]. Since the beginning of the twenty-first century, the number of PLWH has been on the rise and is expected to keep increasing [7].

Aging is often defined as a decline of functional properties at the cellular, tissue and organismal level [8, 9], which increases the vulnerability to disease and mortality [10]. This is of particularly higher concern in PLWH, predisposing to a variety of well-recognized pathologies [11], for instance, atherosclerosis and cardiovascular events [12], loss of renal function [13], osteopenia/osteoporosis [14, 15], and non–AIDS-defining cancers [16, 17]. Increased life expectancy together with HIV-related chronic inflammation and long exposure to some antiretroviral drugs are the main reasons of the accelerated aging in PLWH [18].

However, besides having physical implications, aging also includes psychological and social changes [19–21], conditions that have an evident impact on health-related quality of life (HRQOL). On top of that, PLWH have significantly poorer HRQOL than the general population, despite having a normalized immunological status [22]. Thus, elder people living with HIV (EPWH) combine both aging and HIV-infection features, resulting in ageism, stigma, social isolation and low HRQOL scores [23, 24]. This context brings up new challenges for healthcare professionals, who now have to provide assistance to patients with a significant comorbidity burden and polypharmacy treatments.

While significant resources have been expended on understanding HIV/AIDS pathogenesis and treatment, as well as its impact on individuals, groups, societies and healthcare delivery, so far little research has examined how HIV/AIDS affects the ability of older adults to maintain their daily needs [25, 26]. Opinion of PLWH on the aging process and healthcare attention are not often considered as a variable to study, though this knowledge would help to direct health responses on dissimilar settings. With all this in mind, we attempted to assess the region- and age-specific needs of EPWHOPWH, and their opinion regarding healthcare attention. and compare the differences amongst HIV patients’ needs from different parts of the world.

## Methods

### Study design and population

This is a cross-sectional, multicenter, comparative study including patients aged ≥50 years old or over living with HIV from multiple centers worldwide. A 15- minute - survey was administrated by the clinicians as part of routine carequestionnaire was offered to participants of the study during between the years 2018–2020. Verbal informed consent was obtained prior survey completion, according to Hospital Universitari Germans Trias i Pujol Ethics Committee protocol approval. The investigator informed the patient about the nature and purpose of the study and all participants agreed.

A total of 680 chronic HIV-infected patients were enrolled through health care institutions in Africa (South Africa and Zimbabwe), Central America (Nicaragua, Honduras, Panama and Costa Rica), South America (Argentina, Peru, Colombia, Ecuador and Uruguay), Western Europe (Spain and Italy), and Eastern Europe and Israel (Belarus, Israel, Serbia and Ukraine).

### Study variables and statistical analysis

Enrolled patients were given an in-house, self-completing survey developed by several experts in the HIV field from the participating centers. The questionnaire was divided into two main parts.

The first section was about self-perceived current patient’s situation. It consisted of 24 questions organized as four domains, including general information (health perception, undetectable viral load, serostatus sharing, financial problems, etc), psychological sphere (weariness, depression, loneliness, memory problems), social sphere (having friends and family, active sexual and social life), and specific conditions/geriatric syndromes (sleeping, hearing, visual, mobility and sexual). Affirmative and negative answers were valid. Percentages of affirmative answers were then averaged in order toto get a score for each category, except for the general information section. The second section addressed the patient’s satisfaction about the attention received regarding general medical care, psychological, functional, social, and sexual problems. These questions had either a five-level scale indicating satisfaction, or an open field for the patient to complete. Multiple-choice data were arranged in percent stacked column charts to show satisfaction levels.

Patients were identified in the records by the corresponding code number only. The statistical software used were SPSS 25 for categorical analysis and GraphPad Prism 8 to develop our graphs. A general descriptive analysis of all the variables of the study, overall and separately by 4 regions [Africa, Center and South America (CSA), Western Europe (WE) and South East and Eastern Europe and Israel (SEEEI)] and age groups (50-55, 56-65, 66-75, >75) was assessed by using a Chi-squared test. Finally, by averaging the percentage of affirmative answers in the different sections, we developed three kinds of scores: psychological, social and specific conditions. We sorted these scores by both location and age group and graphed them to visualize tendencies.

## Results

### Cohort description

A total of 680 patients took part in the study, coming from centers across the world in Africa (n = 25; 3.7%), CSA (n = 72; 10.6%), WE (n = 226; 33.2%), and SEEE (n = 357; 52.5%). The majority of participants were male (65.3%), between 56–65 years old (46.6%), and married (40.9%). All these characteristics are summarized in Table 1.

**Table 1:** Summary of patients’ general characteristics

Category		N	%
Region	Africa	25	3.7
	South & Centre America	72	10.6
	Western Europe	226	33.2
	South East and Eastern Europe	357	52.5
Gender	Men	436	65.5
	Women	230	34.5
Age	50–55	243	37.4
	56–65	303	46.6
	66–75	91	14.0
	> 75	13	2.0
Marital status	Married	278	40.9
	Separated/Divorced	131	19.3
	Single	177	26.0
	Widowed	77	11.3

### Patient’s situation

Focusing on the current situation of the patient, at first, a few general aspects on health and habits were recorded. Then, self-perceived psychological, social life and geriatric problems were assessed. Data, sorted by geographical region, are described in detail in Table 2.

**Table 2:** Patient’s situation data sorted by location, expressed as the percentage of affirmative answers

Section	Parameter assessed		Africa	CSA	WE	SEEE	Sig
General information	Median age		54.6	57.4	57.5	60.3	–
	Good Overall health perception		90.0	86.8	73.6	72.5	0.034
	Retired		61.9	35.2	43.8	56.7	0.001
	Exercise	*Minimum*	65.3	66.5	64.8	69.4	0.500
		*Regular*	54.3	46.7	40.2	50.8	0.040
	Regular eating habit		90.5	80.9	93.8	92.1	0.010
	Struggles to remember taking ART		19.0	15.3	9.8	10.7	0.384
	Undetectable viral load		95.2	90.1	96.9	49.1	< 0.001
	Living alone		23.8	31.9	26.5	40.2	0.005
	HIV status sharing		90.5	76.4	63.7	41.9	< 0.001
	Financial problems		90.5	46.4	30.2	45.6	< 0.001
Psychological problems	Weariness		38.9	37.1	45.8	41.3	0.554
	Depression		52.6	25.4	24.5	37.2	< 0.001
	Loneliness		45.0	23.2	22.3	33.1	0.010
	Memory problems		33.3	20.6	36.7	20.4	< 0.001
	Mean score		42.5	26.6	32.3	33.0	**–**
Social sphere	Active social life		71.4	71.8	80.1	49.4	< 0.001
	Active sexual life		35.0	54.2	55.4	46.0	0.069
	Has Friends and Family		85.7	98.6	91.6	73.7	< 0.001
	Mean score		64.0	74.9	75.7	56.4	**–**
Specific/geriatric conditions	Sleep		35.0	38.9	38.5	42.7-	0.702
	Hearing		15.0	21.4	25.2	24.5	0.715
	Visual		73.7	58.3	58.7	48.1	0.017
	Mobility		30.0	16.7	21.3	28.5	0.077
	Sexual		15.0	23.5	21.4	30.3	0.079
	Mean score		33.7	31.8	33.0	34.8	**–**

*CSA* Center and South America, *WE* Western Europe, *SEEE* South Eastern and Eastern Europe

To remark, Africans ranked best on good overall health (90,0%), albeit struggling to remember taking cART (19.0%), being retired (61.9%), and reporting the highest levels of financial problems (90.5%). People in CSA ranked the lowest on being retired (35.2%) and displayed high values on health perception (86.8%) and HIV status sharing (76.4%). Europeans displayed similar levels of health perception, assuming the worst scores (73.6% and 72.5% in the WE and SEEE regions, respectively). WE is the region where subjects describe less financial problems in comparison to others (30.2%). SEEE patients have the lowest percentage of undetectable viral load (49.1%), status sharing (41.9%), and the highest score on living alone (40.2%).

Regarding psychological problems, weariness had a relatively high and consistent prevalence across all regions (from 37.1% to 45.8%). African patients reported the highest on depression (52.6%), loneliness (45.0%), and a very high score on memory problems (33.3%), having a mean score of 42.5%. On the other hand, CSA participants showed the smallest psychological problems mean score (26.6%).

Moving onto the social sphere, patients in Africa and CSA reported a similar level of active social life (71.4% and 71.8% respectively), WE the highest (80.1%) and SEEE the lowest by far (49.4%). Above half of patients in WE and CSA reported active sexual life (55.4% and 54.2% respectively). Finally, almost every patient in CSA (98.6%) and WE (91.6%) reported having friends and family, and SEEE scored the least (73.7%). Overall, the highest social scores were found in WE (75.5%) and CSA (74.9%).

About a third of all patients reported at least one geriatric condition (troubles in sleep, hearing, vision, mobility, or sexual). Sleep and visual problems were relatively high across all regions (over 35%). People in Africa reported the most of visual (73.7%) and mobility problems (30.0%). Patients in SEEE reported the highest on sleep (42.7%) and sexual (30.3%) problems. CSA was the region where the least problems were reported in overall (31.8%).

When we look at the data by age (Table 3), older patients report a better health perception. In this sense, and the oldest EPWH have fewer financial problems (23.1%), more struggles to remember taking ART (23.1%), tend to live alone (69.2%) and share less their HIV status (30.8%). To remark, 39.0% and 46.2% of patients reported financial problems in the 50–55 and 56–65 age groups, respectively.

**Table 3:** Patient’s situation data sorted by age group, expressed as the percentage of affirmative answers

Section	Parameter assessed		50–55	56–65	66–75	> 75	Sig.
General information	Good Overall health perception		81.5	69.9	69.4	91.7	0.008
	Retired		26.9	53.8	98.9	100	< 0.001
	Exercise	*Minimum*	62.9	66.8	68.1	84.6	0.350
		*Regular*	43.1	43.0	45.6	53.8	0.858
	Regular eating habit		89.5	91.3	97.7	92.3	0.129
	Struggles to remember taking ART		13.0	9.9	7.1	23.1	0.207
	Undetectable viral load		78.8	73.0	60.8	63.6	0.020
	Living alone		29.8	34.4	45.1	69.2	0.003
	HIV status sharing		62.0	53.1	40.0	30.8	0.001
	Financial problems		39.0	46.2	34.5	23.1	0.082
Psychological problems	Weariness		39.7	47.4	39.5	30.8	0.207
	Depression		32.7	33.6	27.3	0.0	0.097
	Loneliness		27.7	29.7	31.8	15.4	0.619
	Memory problems		28.0	29.1	16.7	23.1	0.143
	Mean score		32.0	35.0	28.8	17.3	–
Social sphere	Active social life		70.7	58.4	54.4	46.2	0.005
	Active sexual life		58.3	49.7	30.3	7.7	< 0.001
	Has Friends and Family		88.0	80.3	71.4	83.3	0.004
	Mean score		72.3	62.8	52.0	45.7	–
Specific/geriatric conditions	Sleep		38.1	42.8	42.2	50.0	0.638
	Hearing		21.1	24.2	29.7	46.2	0.106
	Visual		59.4	50.7	43.3	69.2	0.025
	Mobility		21.2	24.1	38.2	23.1	0.016
	Sexual		23.0	26.3	32.9	20.0	0.359
	Mean score		32.6	33.6	37.3	41.7	–

*CSA* Center and South America, *WE* Western Europe, *SEEE* South Eastern and Eastern Europe

A pattern of increasing perception of psychological problems is observed since the 66–75 and > 75 groups have the lowest scores on every category. Participants feel less lonely, less depressed and less wearied with increasing age. The 50–55 and 56–65 age categories show the highest score on psychological problems (32.0% and 35.0%, respectively), being weariness its major driver (39.7% and 47.4%, respectively). When we look at the social sphere, we see a pattern of slow decay in social life according to age (from 70.7% to 46.2%, p = 0,005), and an abrupt decay in sexual life (from 58.3% to 7.7%, p < 0.001). In terms of geriatric problems, sleep (50.0%), hearing (46.2%) and visual (69.2%) conditions become worse as the patient ages, but mobility and sexual problems display their peak at ages 66–75 (38.2% and 32.9% respectively). Eldest patients scored the highest on this Sect. (41.7%).

**Fig. 1:**
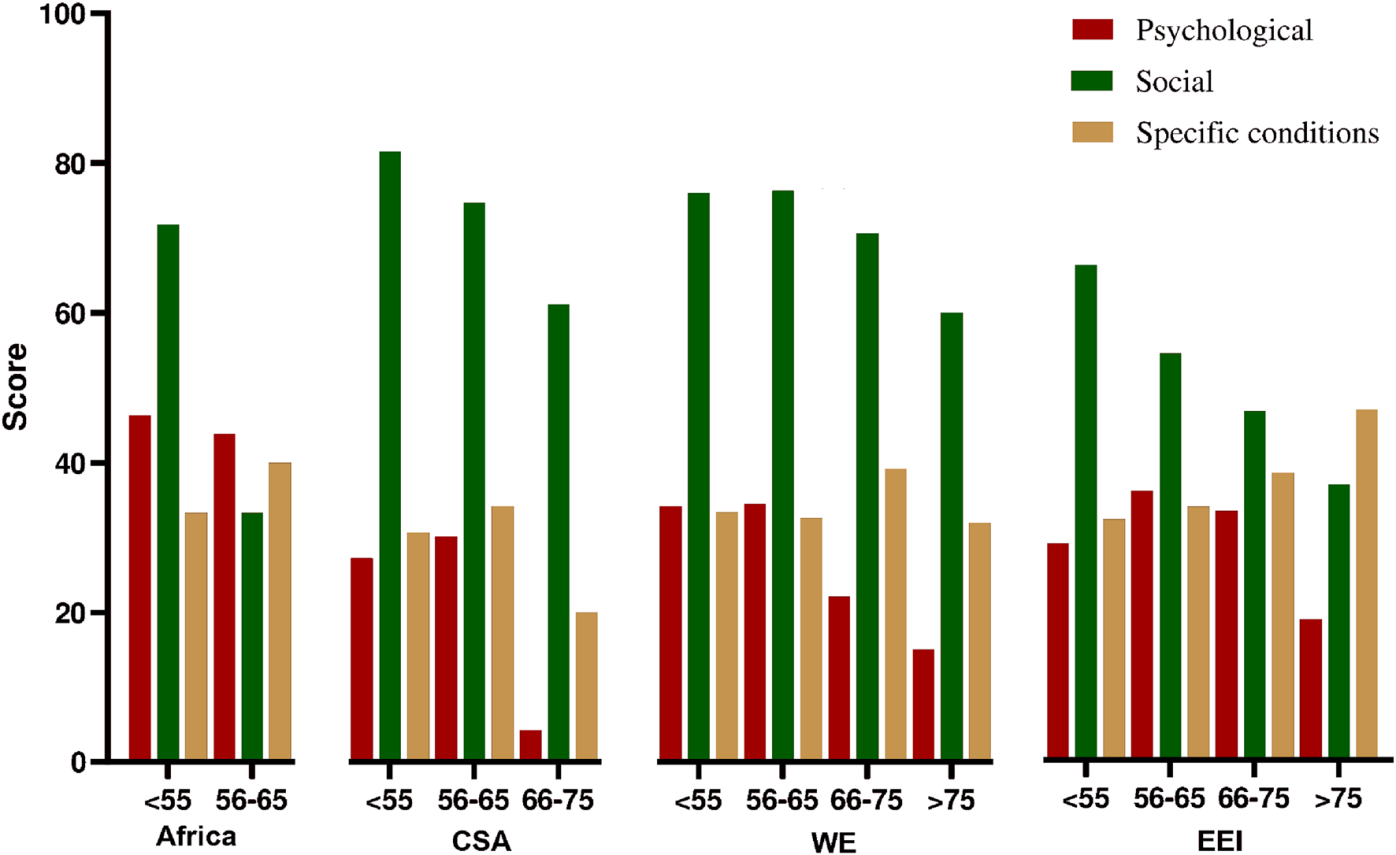
Average section scores sorted by both location and age group. CSA: Center and South America; WE: Western Europe; EEI: Eastern Europe and Israel

Lastly, we developed a psychological, social and specific conditions score by averaging the percentage of affirmative answers in the different sections. We then sorted them scores by both location and age group (Fig. 1). The graph shows a pattern of decay in social sphere and a rise in conditions as people get older.

### Patients’ satisfaction

**Fig. 2:**
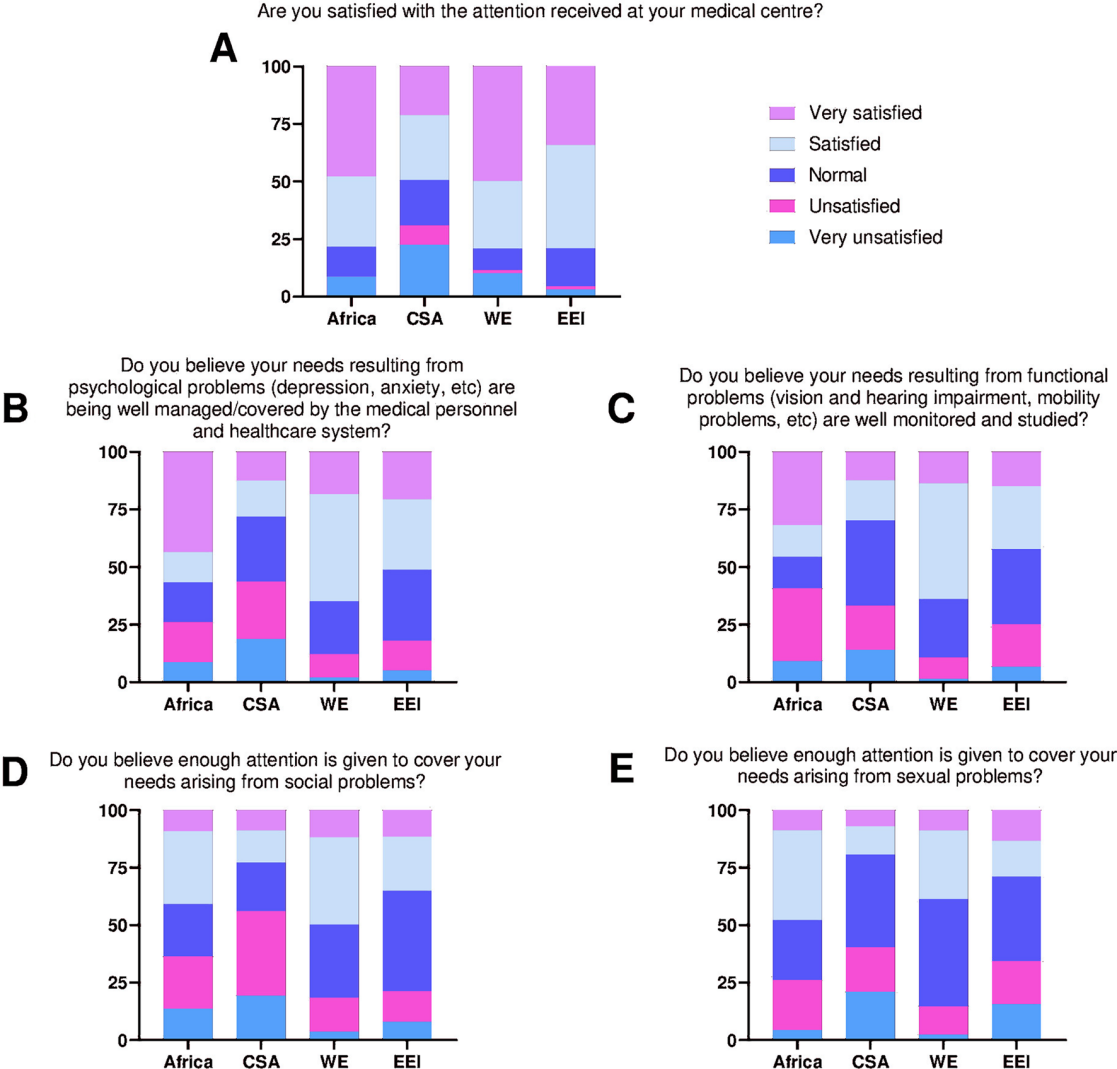
**A:** Patients’ satisfaction about their attention received. CSA: Center and South America; WE: Western Europe; EEI: Eastern Europe and Israel. **B**: Patients’ satisfaction about the management of psychological needs. CSA: Center and South America; WE: Western Europe; EEI: Eastern Europe and Israel. **C**: Patients’ satisfaction about tracking of geriatric conditions. CSA: Center and South America; WE: Western Europe; EEI: Eastern Europe and Israel. **D**: Patients’ satisfaction about social problems assessment. CSA: Center and South America; WE: Western Europe; EEI: Eastern Europe and Israel. **E**: Patients’ satisfaction about sexual problems management. CSA: Center and South America; WE: Western Europe; EEI: Eastern Europe and Israel

The last seven items of the questionnaire addressed the patients’ satisfaction regarding different aspects of their healthcare. Overall, patients were happy with the care received at their medical center – more than 85% of people in Africa and Europe reported so. However, nonsatisfaction (i.e., unsatisfied and very unsatisfied) in this item accounted for ~ 30% of CSA answers (Fig. 2A).

Regarding psychological problems, over 25% of African and CSA participants report nonsatisfaction when asked if they are well managed in this matter, and only few Europeans (~ 15%) felt the same (Fig. 2B).

Over 30% of African and CSA reported nonsatisfaction regarding care of functional problems, while WE showed the best satisfaction scores in this aspect (> 60%) (Fig. 2C). Younger age groups reported higher nonsatisfaction than elder ones across all regions (data not shown). A similar pattern is shared when consultants were asked about needs arising from social problems: ~ 35% of Africans and > 50% of CSA said that these needs were not given enough attention, while less than 20% of Europeans were unsatisfied on this matter (Fig. 2D). Most of nonsatisfied Europeans accounted for > 75-year-old SEEE group (data not shown).

Although nonsatisfaction scores regarding sexual problems are similar to the other aspects, this category shows the worst satisfaction reports (Fig. 2E). In line with this, greater nonsatisfaction scores are seen as the age group is older (data not shown).

**Fig. 3:**
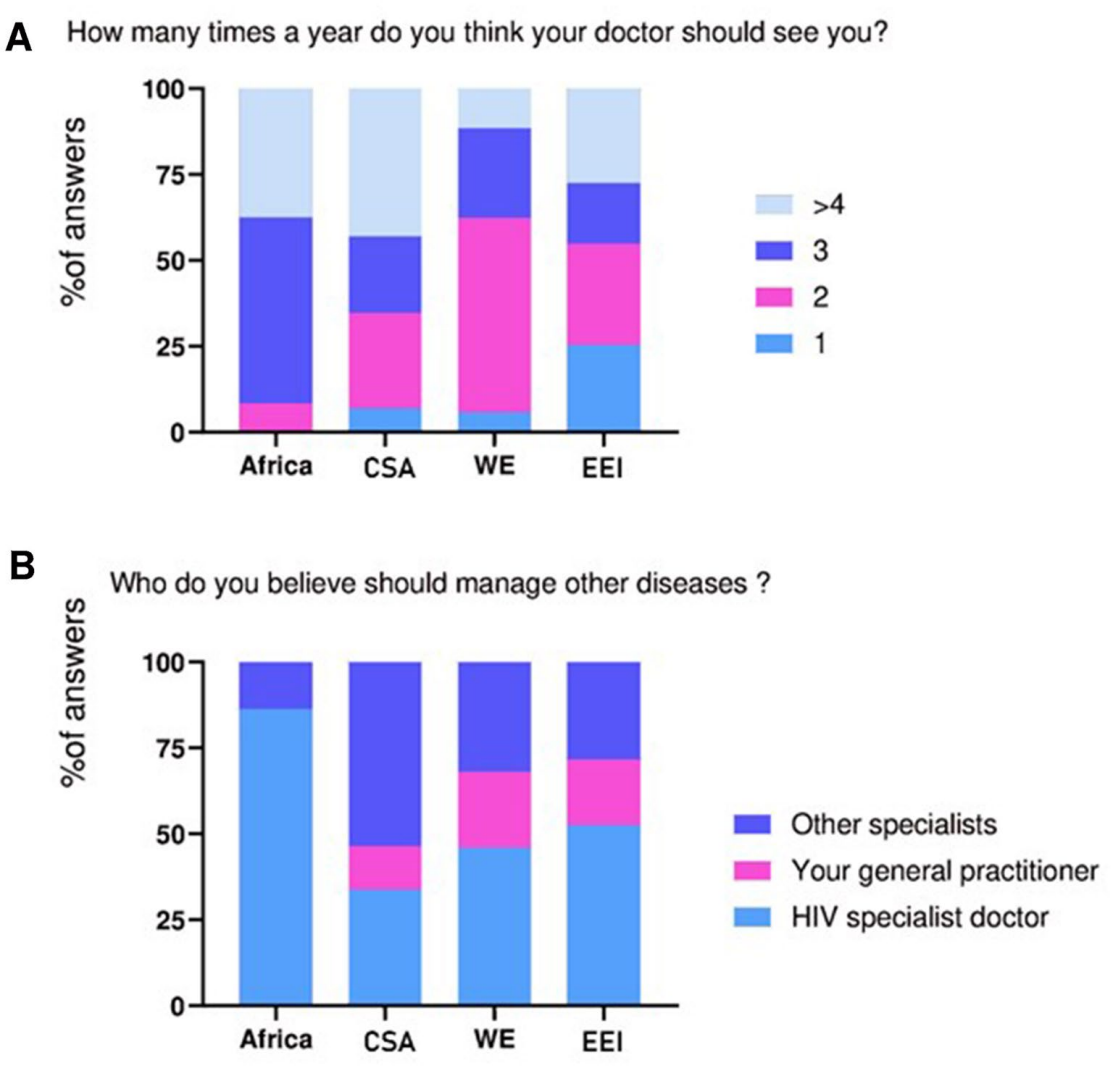
Patients’ opinions on practitioner’s role. CSA: Center and South America; WE: Western Europe; EEI: Eastern Europe and Israel

We then asked our participants two questions regarding their practitioner’s labor. Most Africans and CSA believe their doctor should see them > 3 times a year, while most Europeans said that 1–2 times would be enough (Fig. 3). Interestingly, when asked about management of other conditions, most Africans and nearly 50% of Western and Eastern Europeans preferred their HIV specialist (86%, 46%, and 53% respectively). Over a half of patients in CSA preferred other specialist (54%) (Fig. 3).

## Discussion

Given the efficacy of cART, HIV infection is no longer a life-limiting condition. Nowadays, a special focus needs to be made in addressing the quality of life of the extended life expectancy. Health needs in the elderly are different from that of other age groups, posing clinicians to the additional challenges associated with aging. Patient-generated health data, albeit presenting limitations, is a useful tool to gain insight and improve the care provided by physicians in the aging population worldwide, and thus increase the efficiency of the healthcare system [27]. Here, we assessed the needs of the PLWH aged 50 years or over using a questionnaire collected from patients from different countries. Briefly, our data show relevant differences in the self-perceived patient’s situation and satisfaction with healthcare between regions.

The aging process among PLWH is particular. Together with HIV infection, social circumstances, relationship issues, comorbidities and stigma may have an impact on their HRQOL [23, 28, 29]. For example, according to the Healthy Aging Score criteria developed by the Rotterdam group (an aging score accounting for mental health, cognitive function, physical, social support, and quality of life in general population) [30], a study carried out in OPWH at a tertiary HIV care clinic in Toronto found that only 39% of participants matched the healthy aging range scoring [21]. Following this line, another study found that socio-economic status was significantly correlated to mental and physical HRQOL after adjusting for disease severity in a German cohort of over 700 OPWH [19]. The first stage of our questionnaire consisted of a self-perceived evaluation of the current patient’s situation. Surprisingly, we found that the eldest group (>75 years old) showed the best health perception, despite scoring the highest on geriatric problems and the lowest in the social sphere. This could be explained by considering that these people report a high score in close friends and family and the lowest score in psychological problems and financial problems. In addition, this group reported higher levels of exercise, and that all of them are retired. Of note, this group consisted of only 13 people. Future studies should collect data in OPWH over 65 years old, especially within Africa and CSA to be able to appropriately assess their situation.

It is remarkable that patients in Africa report a great health perception despite a high score in depression, financial problems and loneliness. A similar pattern is seen in CSA, where social problems seem to be central. On the other hand, EEI participants reported the lowest health perception score, probably associated with worse social and loneliness scores. WE show better scores in social and financial problems, but lower scores in health perception.

HRQOL is an essential component of health in PLWH, plus it contributes to other medical outcomes such as HIV infection progression [31]. In line with this, HIV status sharing is a personal decision and has been related to cART adherence, viral load suppression and prevention of HIV infection, particularly disclosure to sexual partners [32, 33]. Supporting previous data, people in EEI report a relatively low social score (56.4%), 40,2% of them live alone, very low HIV disclosure (41,9%) and, an alarming piece of information, less than 50% show undetectable viral load. Social factors might be associated to HIV being a highly stigmatizing, taboo topic, leading to poor ART adherence thus affecting viral load. WE, CSA and Africa show higher sharing scores (63.7%; 76.4% and 90.5%, respectively) and over 90% of them report undetectable viral load. Other remarkable result is that up to a fifth of African participants admitted forgetting taking ART. This could probably be explained by a lack of consciousness about the importance of good adherence, considering other unmet needs. Similarly to others, our results show high rates of poor adherence. A recent meta-analysis of ART adherence on Sub-Saharian Africa yielded that only three fourths of OPWH (>50 years old) are adherent to cART [34]. These are relevant region-specific starting points that highlight where we should strengthen our efforts to.

OPWH are at high risk of geriatric syndromes such as frailty, polypharmacy, and falls [8]. Pre-frailty and frailty affect more than 50% of effectively treated OPWH and is associated with an increased risk of adverse health outcomes that contribute to the overall reduced survival and HRQOL of PLWH [35]. In line with this, as expected, our results show a tendency to develop geriatric conditions as patients are older, with no significant differences among regions. Africa and CSA are the regions with more unsatisfied OPWH, a phenomenon that intensifies in older age groups, although general health perception is better in overall than in Europe. This stands out the needs of these patients for a more comprehensive care. Additionally, sexual activity plays a significant role in the individual’s satisfaction and has often been ignored when analyzing health of older people [36–38]. HIV diagnosis and the fear of transmission have a big impact on sexual life [39, 40], despite the efforts of campaigns such as “Undetectable equals Untransmissible” (U=U). Our findings are in line with literature and highlight the importance of discussing the sexuality of OPWH from an open perspective. A suitable strategy to this would be considering sexual sphere while interviewing our patients.

General care of OPWH should consider both HIV-related and age-related conditions. A Patient-Centered Care [41] approach should be considered as the main strategy, since it attends access to care, emotional support, physical comfort, and respect for patient’s preferences, among other principles. Patient-Centered Care is accomplished by collaborative work between the patient and an interdisciplinary team, which facilitates active patient involvement in decision making [42]. To offer appropriate management, care providers should predict the demands of aging population and change both approach and goals of their offered care [5]. Our results highlight the importance of this matter by showing how the patient needs vary by region and age group. However, needs change from patient to patient, so it is important the implementation of Patient Reported Outcomes (PROs) and the multidisciplinary geriatric assessment of these subjects. Although our study is a screening, it could be considered by care providers to better understand OPWH demands and direct their efforts towards them.

The second part, which addressed for patients’ satisfaction about their healthcare, reflected how different healthcare systems are structured. For instance, in Africa, HIV checkpoints are often more accessible than conventional healthcare. Patients may not need to travel as far, or wait as much as they would in a public hospital. This might be why there was an overwhelming response in African patients when they were asked who should manage their other conditions – 81% of them answered their HIV specialist. On the other hand, people in CSA mostly answered that other specialists should do it. In WE and EEI, half of them prefer their HIV physician, and the other half would think that either their general practitioner or other specialist should do it.

In line with this, people from Africa and CSA are the least satisfied with the healthcare attention they receive for functional, psychological and social problems. Over a half of patients in Africa and CSA would prefer to see their doctor with a higher frequency than every 6 months, whereas most Europeans are content with their situation, and would consider reducing their medical visits to one annually. Again, essential unmet and met needs may be the key to understanding this outcome Our study had several limitations. First, we did not know for how long patients had been living with HIV, which could determine health perception and other psychological and social matters addressed in our study. With only 25 people addressed, Africa is much underrepresented. On top of that, none of those patients was over 65 years old, which means that the needs of African OPWH were not fully measured. Similarly, in CSA there was no data collected for the age group of >75 years old. In fact, only 2% of our participants were over 75 years old, making it difficult to draw strong conclusions on this age group. As this study relies on declarative data, we cannot rule out that some participants may have provided inaccurate details. Finally, these results were collected in 2018, which means that current situation of OPWH may have changed after the COVID-19 burden and the Russian massive invasion on Ukraine, especially in EEI. Indeed, a recent meta-analysis highlighted the effect that the COVID-19 pandemic had in sexual heath, including disruptions in HIV/STI testing, and changes in sexual behaviors [43]. Prospective studies should be carried out since they allow us to understand a wider scope of needs among HIV aging population, as well as the determining factors to improve treatment results based on patients’ needs.

## Conclusions

Targeting care and services for the HIV aging population should be based on individual patients’ needs. OPWH needs differ depending on both geographical location and age. In EEI, we found that socializing HIV and reaching undetectable viral load would be the main goals. CSA participants are the least satisfied regarding their healthcare, which could be due to higher expectations or profound inequities in the region. Africans show the best health perception, and their satisfaction reports mirror a system where general health is hard to achieve, but international aid on HIV-related problems is much more appealing to them. WE is the most developed region studied and their participants report the best satisfaction scores.

On top of that, our study data emphasizes that this population requires functional, psychological, and emotional support overall. Age-specific information, education and counseling programs are needed to promote physical and mental health among older adults living with HIV/AIDS. This is crucial for improving HRQOL and patient’s satisfaction. The combined management of HIV/AIDS and age-related conditions will have a profound impact on an already challenged health care delivery system. The need for caregiving and its critical role in health care management is most evident in older adults. The primary goal of research and treatment during the first two decades of the HIV/AIDS epidemic was to keep patients alive and healthy with innovative therapies. Now, it is time to focus on the quality of that extended life that older HIV-infected people have achieved.

## Data Availability

All data and materials support our published claims and comply with field standards.
